# German Farmers' Awareness of Lameness in Their Dairy Herds

**DOI:** 10.3389/fvets.2022.866791

**Published:** 2022-03-24

**Authors:** Katharina Charlotte Jensen, Andreas W. Oehm, Amely Campe, Annegret Stock, Svenja Woudstra, Melanie Feist, Kerstin Elisabeth Müller, Martina Hoedemaker, Roswitha Merle

**Affiliations:** ^1^Department of Veterinary Medicine, Institute for Veterinary Epidemiology and Biostatistics, Freie Universität Berlin, Berlin, Germany; ^2^Clinic for Cattle, University of Veterinary Medicine, Foundation, Hannover, Germany; ^3^Clinic for Ruminants With Ambulatory and Herd Health Services, Ludwig-Maximilians Universität Munich, Munich, Germany; ^4^Department of Biometry, Epidemiology and Information Processing (IBEI), WHO Collaborating Centre for Research and Training for Health at the Human-Animal-Environment Interface, University of Veterinary Medicine Hannover, Foundation, Hannover, Germany; ^5^Clinic for Ruminants and Swine, Faculty of Veterinary Medicine, Freie Universität Berlin, Berlin, Germany

**Keywords:** locomotion score, personality trait, Farmers' Detection Index, attitude, claw health, mobility score

## Abstract

Lameness is one of the most challenging problems in the dairy industry. Control is impeded because farmers often underestimate the number of lame cows. The objectives of this study were to assess German farmers' awareness of lameness in their herds and to determine the associations between farmers' awareness and their management practices, farm characteristics as well as with farmers' education, personality traits and attitudes. As a part of a large cross-sectional study, veterinarians visited farms in three structurally different regions of Germany: north (*n* = 253), east (*n* = 252), and south (*n* = 260). The cows (*n* = 84,998) were scored for locomotion and farmers were asked to estimate the number of cows that were lame or did not walk soundly. The ratio of farmers' estimated prevalence and the veterinarians' observed prevalence (Farmer's Detection Index; FDI) was calculated. The median lameness prevalence assessed by the veterinarians was 23.1, 39.1, and 23.2%, and the median prevalence of lame cows estimated by the farmers was 9.5, 9.5, and 7.1% in the north, east, and south, respectively. On average, farmers were conscious of only 45.3% (north), 24.0% (east), and 30.0% (south) of their lame cows. Farmers managing their herds according to organic principles had a higher FDI than farmers who managed their herds conventionally. Surprisingly, no significant associations between FDI and factors concerning claw health management could be detected. Therefore, increased awareness did not seem to be necessarily linked to improved management. Moreover, the FDI was not significantly associated with farmers' education or herd size. In the south, more extraverted farmers had a lower FDI. Those farmers who totally agreed with the statement, “I am satisfied with my herd's health,” had a lower FDI than farmers who disagreed or were undecided. Moreover, farmers who disagreed or were undecided with the statement, “It affects me to see a cow in pain” had a higher FDI than those farmers who agreed to the statement. The results indicate that poor awareness of lameness was linked to the farmers' attitude and personality. Therefore, new approaches concerning the consultation regarding lameness control, such as the use of Motivational Interviewing, might be useful in the future.

## Introduction

Lameness is a serious problem in the dairy industry worldwide. It is known to compromise animal welfare (1) and to lead to economic losses (2, 3). Lameness has gained increasing attention in the dairy sector, and numerous studies have identified several risk factors (4–6). However, lameness prevalence in dairy herds remains high. In the UK, the mean within-farm prevalence increased from 20.6% in 1989 to 1991 (7) to 36.8% in 2006 to 2007 (8), and was estimated to be at 31.6% in the years 2015 to 2016 (9). In other countries around the world, a similar or slightly lower prevalen ce has been observed (10–12).

Farmers frequently underestimate the percentage of lame cows within their herds (13–17), as well as the financial consequences (13). To date, there has been limited understanding of why farmers have a poor awareness of lameness in their herds. In a study by Leach et al. (13), the estimation of farmers corresponded to the number of cows diagnosed as severely lame by the researchers. It was hypothesized that farmers overlook cows with moderate lameness and often have another definition of “lameness.” This finding is supported by the observation that farmers avoid the term “lame” and prefer “impaired mobility” (18). A second hypothesis for the underestimation is that individual cows gain less attention from farmers (13) due to increasing herd sizes and other tasks apart from the stable, like, e.g., documentation.

Raising awareness is the first step in improving lameness in dairy herds (19). Farmers who stated that lameness was a major problem had a lower lameness prevalence in their herds than farmers who stated otherwise (14). This finding may appear surprising. However, as long as farmers are not aware of the lameness status of their herd, they are not likely to implement measures to improve the situation.

Although numerous studies have shown an underestimation of the proportion of lame cows by farmers, knowledge about how the awareness can be increased is limited. It is known that the personality and attitude of farmers are connected to their willingness for behavior change (20, 21). To further improve lameness prevention and control in dairy herds it is therefore necessary to better understand the connection of the farmers personality and the awareness of lame cows within their herds. Moreover, poor knowledge exists if the awareness differs between various types of farms and if it is associated with an improved or intensified claw health management. Therefore, the objectives of this work were to first describe farmers' awareness of lameness in their dairy herds in three structurally different regions in Germany. Secondly, this study aimed to determine which factors related to farm structure, claw health management, farmers' attitudes, education, and personality, were associated with the farmers' detection rate of lameness.

## Materials and Methods

### Study Region

This study was conducted as part of a large cross-sectional study on the health, biosecurity, and housing environment of dairy farms in Germany (22). The project was initiated and funded by the German Federal Ministry of Food and Agriculture (BMEL) through the Federal Office for Agriculture and Food (BLE).

The study was carried out in three regions of Germany with different structures of the dairy industry: in the north (including the federal states of Schleswig-Holstein and Lower Saxony), dairy farms are mostly family-run businesses with a mean herd size of 95 cows in Lower Saxony and 102 cows in Schleswig-Holstein (23). The east includes most parts of the former German Democratic Republic (federal states: Mecklenburg-West Pomerania, Brandenburg, Thuringia, Saxony-Anhalt). Here, mean herd sizes vary between 184 and 230 cows (23) and farms are partly run by cooperations. Most of these farms engage several employees. All farms in the south were located in Bavaria. There, dairy farms are small, mostly family-run businesses, and partly run part-time. Mean herd size is 42 cows (23).

### Recruitment of Farms and Farm Visit

Veterinarians visited 253 (north), 252 (east), and 260 (south) farms on a single occasion between December 2016 and July 2019. Farmers knew that a mobility scoring would be performed during this visit. Farms were recruited continuously during the study period to offer farmers a prompt date for the farm visit. The sample size was calculated as described by Dachrodt et al. (22). In brief, in the north and east, farms were randomly selected from the complete list of dairy cattle owners in the National Traceability and Information System for Animals (Herkunftssicherungs- und Informationssystem für Tiere); in the south, they were randomly selected from dairy farmers organized in the neutral auditing organization for Bavarian dairy farms (Milchprüfring Bayern e.V.). Based on the number of milking cows within each region, the farms were divided into three groups based on herd size to gain an equal distribution over all farm sizes. A separate sample size was calculated for every region, stratified by herd size and federal state (22). Selected farms received invitations to participate. Farmers voluntarily contacted the study teams via telephone, email, or postal mail, and a date for a farm visit was arranged. The participation rate ranged between 6 and 9%, depending on the region.

### Locomotion Scoring

On the occasion of the farm visit, locomotion scoring was performed, including lactating and dry cows. In free-stall barns, straw yards, and pastures, locomotion scoring was performed according to Sprecher et al. (24). This score ranges from 1 (normal gait) to 5 (severely lame). Whenever cows were kept tied and did not have access to pasture on the day of the farm visit, locomotion scoring was performed using the Stall Lameness Score [SLS; (25)]. In larger herds, a randomly selected sample size was scored: in the north, with up to a herd size of 213 cows, all cows were scored. In larger herds, at least 213 randomly selected cows were assessed per farm. In the east, 166 cows were scored in herds between 166 and 292. In smaller farms, all cows, and in farms with more than 292 cows, 292 cows were scored. In the south, a maximum of 130 cows were scored. The calculation of the sample sizes was based on an assumed lameness prevalence of 40%, a confidence level of 95%, a power of 80%, and the different cut-offs for small, medium and large farms in the three regions. If a sample was scored and the farm had more than one pen for cows, the observers selected a similar percentage of cows in each pen. If for instance, 80% of a herd had to be scored, the veterinarians scored four cows standing next to them and marked a fifth cow without keeping records of this cow.

In total, 21 veterinarians assessed the locomotion of cows. They were trained using photos, videos and SOPs. Telephone conferences were held to discuss open questions—first weekly, later as required. To safeguard the reliability of the observations, veterinarians met on three different occasions during the study and inter-observer agreement was assessed by performing locomotion scoring on the same 36, 53, and 20 cows.

### Interview and Further Data Collection

A face-to-face, pen-and-pencil interview was conducted with the farmer or herd manager (following referred to as “farmer”). The farmer was asked to give an estimation of the number or the percentage of dry and lactating cows in their herd that were lame or “did not walk sound.” The interview was usually performed prior to or simultaneously with the scoring of the herd, so that the farmer did not gain any further information from the scoring. However, they were aware that veterinarians would perform locomotion scoring on the same day. Furthermore, farmers answered questions about additional aspects, such as their attitude, education, and management of their dairy farm.

After the interview, the veterinarian handed a brief HEXACO questionnaire to the farmer. The HEXACO questionnaire is an item-centered approach used to assess personality traits. We used the 24-item Brief HEXACO Inventory (26). Due to concerns regarding the acceptance of farmers, the items of the domain honesty-humility were removed. Therefore, the questionnaire consisted of 20 statements assessing the five traits emotionality, extraversion, agreeableness, consciousness, and openness to experience. Farmers rated their consent to each statement on a five-point Likert scale (“strongly disagree,” “disagree,” “neutral,” “agree,” “strongly agree”). The farmer was asked to fill it out on their own and hand it back in a closed envelope.

Housing conditions were assessed using protocols. These protocols as well as the questionnaires are available online (https://ibei.tiho-hannover.de/praeri/uploads/report/PraeRi_Anhang3_Handbuch.7z and https://ibei.tiho-hannover.de/praeri/uploads/report/PraeRi_Anhang2_EB_FB.7z; only in German language). If available, data from the monthly milk recording (DHI) were included in the relational study database.

### Statistical Analyses

The statistical unit was the farm. The three study regions were analyzed separately due to the structural differences in dairy industry.

#### Lameness Prevalence and Farmers Awareness

A cow was considered lame with a locomotion score according to Sprecher et al. (24) of 3 or more or if she displayed two or more criteria of the SLS. As aforementioned, farmers may have another definition of “lame cow” and experience cows as lame when they are in fact severely lame. Therefore, the prevalence of lameness and severe lameness (score 4 or 5) was calculated (veterinarians' prevalence; VP/ VP_severe). On some farms, not all cows could be scored based on their locomotion. Herds with more than 10% missing values were excluded from further analyses.

In cases where the farmer stated a number of cows, the estimated prevalence for the farmer was calculated by dividing the number by the herd size. If the farmer stated a percentage, this number was defined as the farmers' prevalence (FP). The agreement of FP with VP and VP_severe was described using Lin's concordance correlation coefficient [CCC; (27)] and Spearman's correlation coefficient, respectively. The quotient of FP and VP, called Farmers' Detection Index [FDI; (28)], described the percentage of the lame cows the farmer was aware of.

#### Factors Associated With Farmers' Awareness

Concerning the second objective, the following factors were assessed as follows:

(1) farm characteristics:
  - herd size (sum of lactating and dry cows; source: DHI or interview)
  - culling rate due to lameness (number of cows culled due to lameness during the 12 months prior to the farm visit divided by herd size; source: questionnaire)
  - milk yield (mean milk yield (kg) per cow and year; source: DHI or if unavailable, questionnaire)
  - housing system (more than 80% of the cows in one of the following systems: tie stalls, pen with cubicles, straw pen, pasture, or mixed (<80% of cows in one system); source: scoring)
  - Husbandry (conventional or organic; source: questionnaire)

(2) claw health management (source: questionnaire):
  - use of a veterinary herd health management program (VHHMP; no participation, participation in a general VHHMP, participation in a VHHMP including claw health)
  - detection of lameness (no lameness detection, lameness detection during stable work, or lameness detection as a separate work task)
  - person performing the claw trimming (farmers themselves or professional claw trimmer/veterinarian)
  - frequency of regular claw trimming events/year (once a year or less, twice a year, or three times a year or more often)

(3) attitude and education (source: questionnaire):
  - highest professional education (no professional education as a farmer, agricultural apprenticeship, further agricultural education, or university degree in agricultural science)
  - further education (agreement with the statement “I regularly attend technical seminars.”)
  - satisfaction with herd health (agreement with the statement “I am satisfied with my herd's health.”)
  - relationship toward cows (agreement with the statement “I have an emotional relationship to my cows.”)
  - empathy toward cows (agreement with the statement “It affects me to see a cow in pain.”)
  - mental workload (agreement with the statement “My job as a farmer puts weight on me.”)
  - claw health as a breeding goal (yes/no)

(4) personality (source: HEXACO-questionnaire; median of the depending items):
  - Emotionality
  - eXtraversion
  - Agreeableness
  - Conscientiousness
  - Openness

First, descriptive analyses were conducted. The FDI was not normally distributed, and log transformation did not result in a normal distribution of residuals when using linear regression. Therefore, due to the explorative nature of the analyses, the associations between FDI and the factors were calculated using Spearman's correlation coefficient for quantitative factors and Kruskal-Wallis tests for categorical factors.

Owing to the large number of factors, multiple testing existed due to the second objective. The *p*-value of 0.05 was divided by the number of variables (*n* = 21). Therefore, significance level concerning the associations was assumed if *p* < 0.0024 [Bonferroni method; (29)].

For statistical analyses, SAS 9.4© (SAS Institute Inc., Cary, North Carolina 27513, USA) was used. Figures showing the distribution of FP, VP, and FDI were created using R 4.0.3 (“Bunny-Wunnies Freak Out”) using the R Studio interface and the packages *ggplot2* (30) and *dplyr* (31).

## Results

### Observer Agreement and Study Population

Three meetings between the observers were conducted during the study period. The weighted kappa measuring the agreement between the observers concerning the ordinal locomotion score according to Sprecher et al. (24) varied between 0.4 and 0.6, indicating a moderate agreement (32).

Milk yield, herd size, and average age of cows in the herds are shown in Table 1. As expected, herds in the south were much smaller, and the milk yield was lower than that in the other regions. The average age of the cows in the herd was lower in the east than in the other regions. In the north and east, Holstein Friesian was the predominant (>80% of cows in the herd) breed: 90.1 and 86.1%, respectively. In the south, 55.4% of the farmers mostly kept Simmental, and only five farmers (1.9%) preferred Holstein Friesians the most. The other farmers in the south kept other breeds (7.3%) or a mix of breeds (35.4%).

**Table 1:** Characteristics of farms.

**Region**	***N***	**Mean**	**Min**	**25%-Q**	**Median**	**75%-Q**	**Max**	**CV**	**Missing**
**Mean milk yield per cow and year (kg)**
North	240	9,062	3,597	8,080	9,330	10,068	12,069	15.3	13
East	248	9,222	2,739	8,543	9,481	10,299	12,907	17.8	4
South	231	7,606	3,712	6,987	7,706	8,456	10,598	16.1	29
**Herd size (number of dry and lactating cows)**
North	253	104.6	12	59	87	127	1,011	84.5	0
East	252	355.2	1	132.5	254.5	459.5	2,952	102.7	0
South	260	45.0	4	25.5	40	59	247	67.9	0
**Average age within the herd (years)**
North	240	4.7	3.1	4.3	4.7	5.0	7.4	11.9	13
East	247	4.4	2.9	4.1	4.3	4.7	7.7	14.2	5
South	231	4.9	3.8	4.4	4.8	5.2	7.4	13.1	29

Locomotion scoring was performed on 85,549 cows from 765 farms (north: 253; east: 252; and south: 260). Scoring data from 14 farms were excluded from further analyses due to missing data. Finally, 84,998 cows (north: 24,395; east: 49,675; south: 10,928) from 751 farms (north: 251; east: 251; south: 249) were included in further analyses. Except for three farmers, all estimated the proportion of lame cows within their herds.

### Lameness Prevalence and Farmers' Awareness

**Figure 1:**
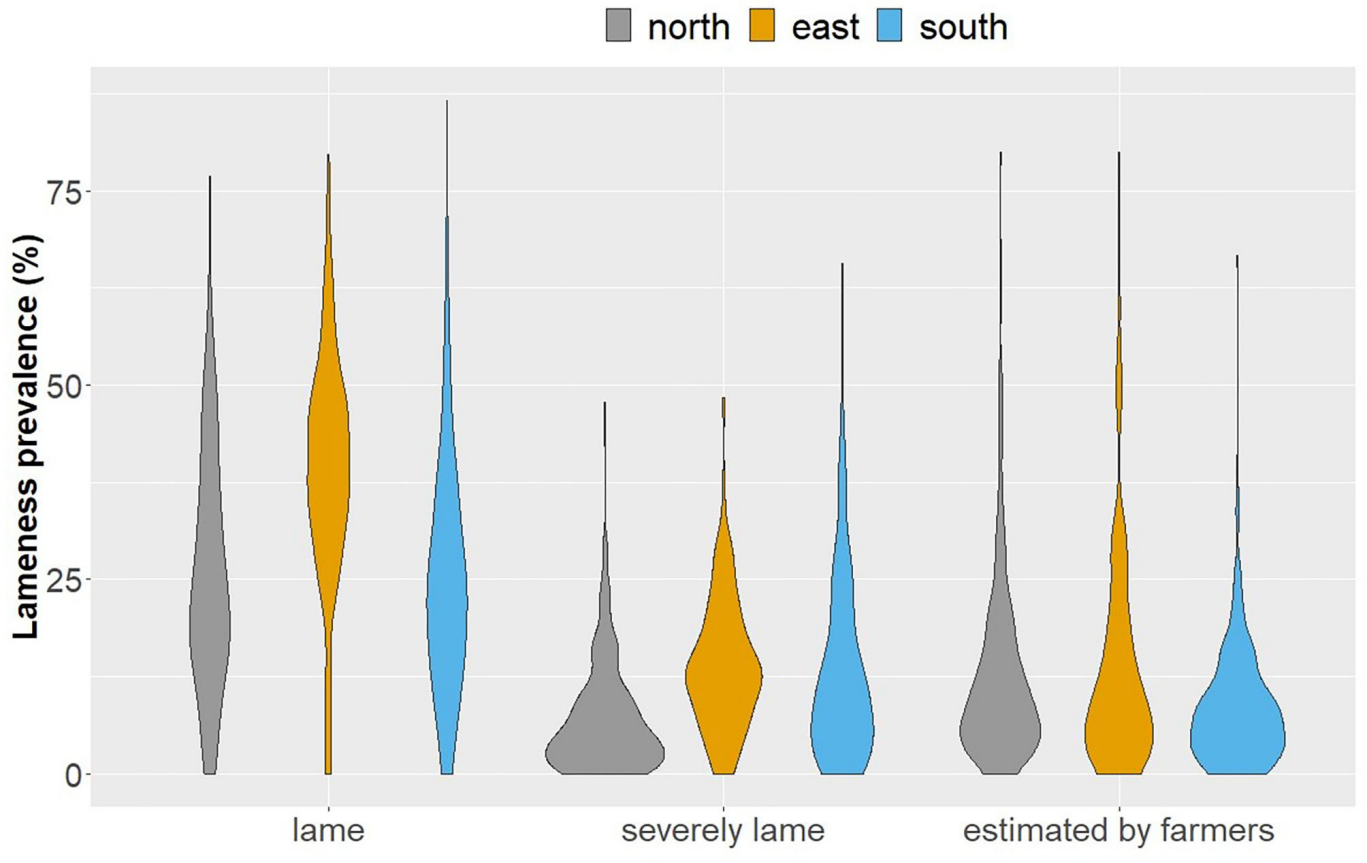
Distribution of lameness prevalence assessed by researchers and estimated by farmers grouped by region. Researchers' prevalence: *n* = 251 (north and east), *n* = 249 (south). Farmers' prevalence: *n* = 253 (north), *n* = 250 (east), *n* = 259 (south).

VP, VP_severe, and FP are shown in Figure 1. The percentages classified as lame by the study veterinarians were similar in the north and south (Q25/Q50/Q75: north, 14.9/23.1/34.9%; south, 14.4/23.2/33.3%), but higher in the east (30.9/39.1/47.8%). Twenty-seven of 757 farms (3.6%) had no lame cows (north: 9 farms [3.6%], east: 7 farms [2.8%], south: 11 farms [4.4%]).

The estimated lameness prevalence by the farmers was below 10% on average (Figure 1; Q25/Q50/Q75: north, 4.8/9.5/16.1%; east, 3.8/9.5/20.0%; south, 3.3/7.1/11.5%). Fifty-eight of 762 farmers (7.7%) estimated no lame cows (north: 11 farmers [4.4%], east: 15 farmers [6.0%], south: 32 farmers [12.4%]). Of these farmers, 20 really had no cows. Twelve of these 58 farmers had a VP of ≥ 25%. Seven farmers stated to have at least one lame cow, but no lame cows were observed. The FDI could not be calculated for the farmers with no lame cows.

The agreement between FP and VP is presented in Table 2. A slight to fair agreement was achieved (32). The agreement between FP and VP_severe was slightly higher (Table 2).

**Table 2:** Agreement of the lameness prevalence estimated by the farmer (FP) and the prevalence of lame (VP) and severely lame (VP_severe) cows assessed by the researchers.

		**North (*n* = 251)**	**East (*n* = 249)**	**South (*n* = 248)**
Agreement of FP and VP	CCC (Lin)	0.34	0.16	0.14
	CC (Spearman)	0.52	0.41	0.31
Agreement of FP and VP_severe	CCC (Lin)	0.33	0.34	0.24
	CC (Spearman)	0.45	0.45	0.31

*CCC (Lin), Lin's Concordance Correlation Coefficient*.

*CC (Spearman), Spearman's Correlation Coefficient*.

**Figure 2:**
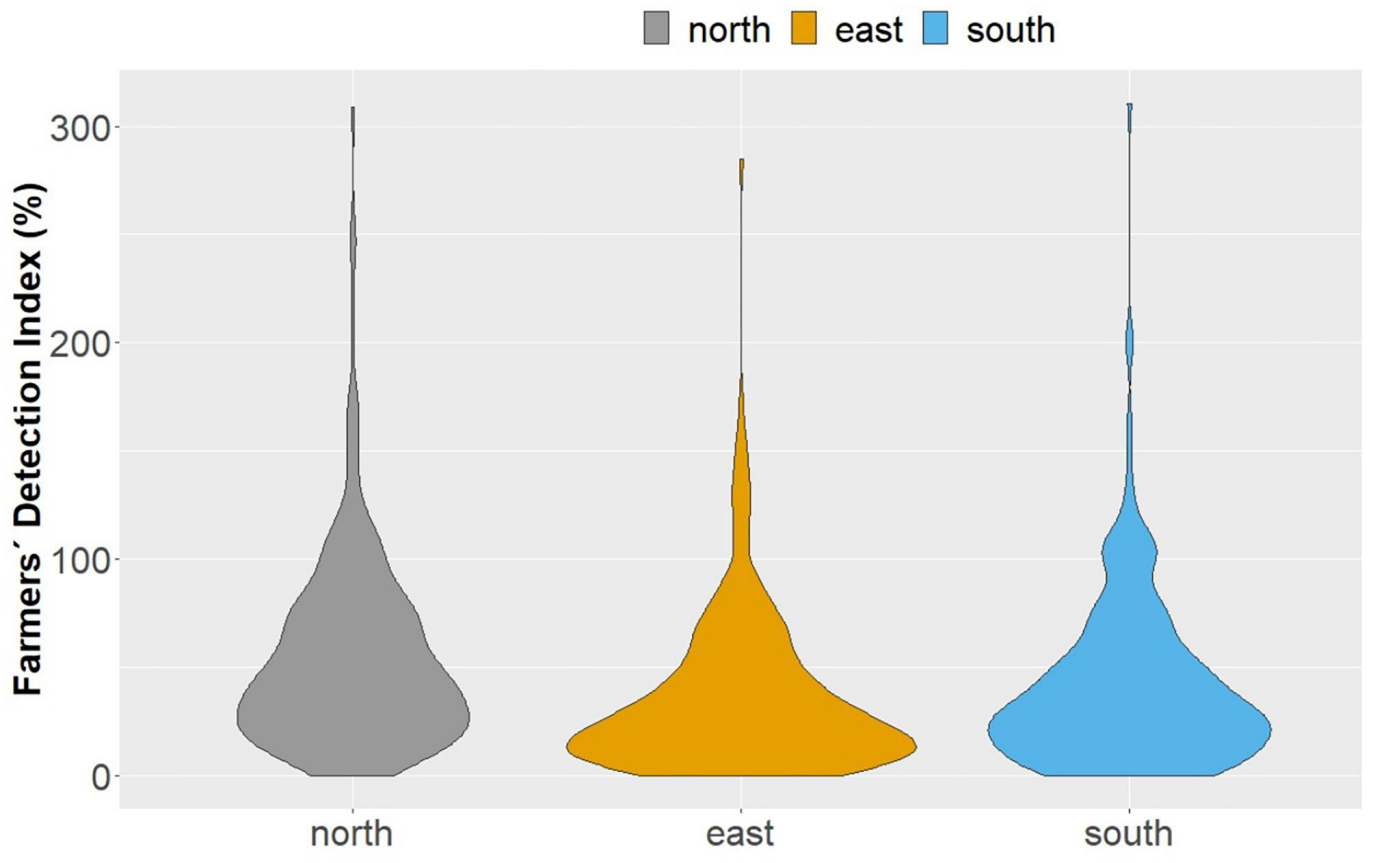
Distribution of Farmers' Detection Index (% of lame cows estimated by farmer/ % of lame cows assessed by researchers*100) by region *n* = 242 (north and east), *n* = 237 (south).

The underestimation of lame cows was corroborated by low FDI (Figure 2). On average, farmers were conscious of every second to fourth lame cow (Q25/Q50/Q75: north, 22.9/42.9/73.3%; east, 11.2/24.0/49.7%; south, 14.8/30.0/53.9%). However, 62 farmers also overestimated the proportion of lame cows in their herds (FDI > 100: north, 30; east, 14; south, 18 farmers). The half of this group overestimated the prevalence only slightly by up to 20%, but the other half of this group overestimated the percentage by more than 20% and nine farmers stated to have two-times more lame cows than the researchers observed.

### Factors Associated With the Farmers' Awareness

The associations between farm characteristics, claw health management, farmers' education, attitude, personality, and FDI are shown in Tables 3–**7**.

**Table 3:** Association (Kruskal-Wallis-test) and descriptive analyses of Farmers' Detection Index depending on farm characteristics (qualitative variables).

	**North**				**East**				**South**			
	***N***	**25%Q**	**Median**	**75%Q**	***N***	**25%Q**	**Median**	**75%Q**	***N***	**25%Q**	**Median**	**75%Q**
Main housing system^*^
Tie stalls	5	0.0	10.0	43.5	3		0.0		63	0.0	33.3	57.4
Cubicles	210	22.9	41.6	72.6	189	12.6	26.3	52.2	167	15.8	30.0	54.9
Straw pens	6	49.1	82.1	210.3	8	10.4	13.1	52.1	1		0.0	
Pasture	5	50.0	52.2	100.0	3		0.0		0			
Mixed	16	26.4	64.2	97.4	39	8.8	19.1	42.7	6	5.8	17.0	36.6
*P*-value				0.1092				0.7365				0.8071
**Farming**
Conventional	232	22.5	41.9	71.7	217	11.5	24.1	47.4	180	13.9	28.6	51.6
Organic	10	75.7	84.1	97.2	15	10.1	28.9	62.6	34	17.2	40.2	77.9
*P*-value				0.0152				0.8457				0.0461

*Farms within categories with n < 5 were not incorporated in Kruskal-Wallis-test*.

* *≥80% of cows were kept in this system at the day of the farm visit; mixed implies that not 80% were kept in one system but different systems were used*.

Regarding the farm characteristics (Tables 3, 4), farmers working according to organic principles had a higher FDI in the north and south (north, *p* = 0.0152; east, *p* = 0.8457; south, *p* = 0.0461). In the east, farmers who stated that they had a higher percentage of cows culled due to lameness had a higher FDI (*p* < 0.0001). Significant associations between milk yield or the housing system and FDI could not be detected. In the south, lower FDI was observed in larger herds (*p* = 0.0400). This association was not observed in the other regions where the herds were generally larger.

**Table 4:** Spearman's correlation analyses: Farmers' Detection Index and farm characteristics (quantitative variables).

	**North**			**East**			**South**		
	***N***	**SCC**	***P*-value**	***N***	**SCC**	***P*-value**	***N***	**SCC**	***P*-value**
Herd size	242	−0.07	0.3064	242	0.09	0.1577	242	−0.13	0.0400
Milk yield (kg)	233	−0.06	0.3502	239	0.06	0.3536	215	−0.06	0.3958
Culling rate due to lameness	238	0.06	0.3651	242	0.26	<0.0001	236	0.04	0.5058

*SCC, Spearman's Correlation Coefficient*.

Surprisingly, there were no significant associations between the factors of claw health management and FDI (Table 5).

**Table 5:** Association (Kruskal-Wallis-test) and descriptive analyses of Farmer' Detection Index depending on claw health management.

	**North**				**East**				**South**			
	***N***	**25%Q**	**Median**	**75%Q**	***N***	**25%Q**	**Median**	**75%Q**	***N***	**25%Q**	**Median**	**75%Q**
**Participation in veterinary herd health management program (VHHMP)**												
No participation	110	22.2	42.7	72.6	95	10.1	21.0	49.7	192	16.5	30.0	53.5
Participation in general VHHMP	90	22.5	46.3	79.2	65	11.6	35.2	51.8	41	11.1	22.7	54.5
Participation in VHHMP incl. claw health	42	23.6	39.7	67.6	81	12.5	37.5	45.4	3		29.0	
*P*-value	0.5662				0.6759				0.8970
**Mean frequency of claw trimming per cow**												
Once a year or less	59	20.6	39.9	71.4	20	6.3	16.1	30.9	90	12.0	31.9	53.9
Twice a year	140	23.6	44.9	73.9	127	14.8	26.0	53.0	131	16.2	29.0	50.9
Three times a year or more often	40	22.5	41.9	78.3	93	8.8	21.5	46.2	11	10.3	51.6	75.0
*P*-value				0.6061				0.0430				0.8772
**Person who mainly performs claw trimming**												
Farmer	52	25.4	39.0	71.2	26	11.3	20.7	53.0	103	15.8	32.8	54.9
Claw trimmer or veterinarian	190	22.2	43.7	74.4	216	11.4	24.3	49.0	132	14.5	28.6	54.2
*P*-value				0.8977				0.8228				0.6619
**Lameness detection (LD)**												
No LD	9	18.8	29.7	80.1	6	21.3	40.1	52.4	14	9.1	29.4	50.0
LD during stable work	226	23.7	43.2	74.4	217	11.2	23.0	48.3	216	14.8	29.7	54.2
LD as a separate work task	7	17.5	28.0	56.8	19	15.4	27.6	65.1	6	33.3	46.4	66.7
*P*-value				0.4974				0.3302				0.6688

In the north and south, farmers with a university degree in agriculture had a higher FDI than farmers with another education (Table 6).

**Table 6:** Association (Kruskal-Wallis-test) and descriptive analyses of Farmers' Detection Index depending on farmer's attitude and education.

	**North**				**East**				**South**			
	***N***	**25%Q**	**Median**	**75%Q**	***N***	**25%Q**	**Median**	**75%Q**	***N***	**25%Q**	**Median**	**75%Q**
**Highest degree of professional education**
No professional education as a farmer	23	20.9	50.0	91.0	15	0.0	8.7	30.2	30	8.1	29.9	50.0
Agricultural apprenticeship	45	18.8	39.4	72.0	40	12.9	24.2	45.4	93	13.0	28.6	53.9
Further agricultural education^a^	151	22.7	42.2	71.1	41	11.6	27.6	53.8	88	17.3	29.0	50.80
University degree in agricultural science	23	23.7	55.8	100.0	146	12.1	24.8	55.3	10	20.7	50.4	64.4
*P*-value				0.7018				0.0297				0.6123
**“I regularly attend technical seminars.”**
I (totally) disagree	28	18.7	35.9	76.5	15	6.9	12.1	55.1	22	9.1	23.7	50.0
Neutral	24	29.3	56.8	80.4	24	6.5	20.6	31.9	31	14.8	33.3	77.9
I agree	107	25.5	46.4	74.4	73	10.5	20.3	42.7	88	20.3	29.9	54.4
I totally agree	80	18.6	39.7	71.7	60	11.3	22.7	42.8	96	12.5	25.9	53.0
*P*-value				0.3328				0.6682				0.4846
**“I am satisfied with the health of my herd.”**
I (totally) disagree	46	36.4	51.8	80.0	43	10.5	20.3	53.8	30	14.8	29.5	52.7
Neutral	47	21.5	38.6	79.2	35	17.8	32.7	49.9	42	23.1	38.3	67.8
I agree	125	22.5	46.4	74.9	84	9.1	20.1	35.0	138	15.7	32.2	54.5
I totally agree	20	16.7	24.5	43.3	10	0.0	5.0	15.4	27	0.0	16.2	50.0
*P*-value				0.0175				0.0027				0.0191
**“I have an emotional relationship to my cows.”**
I (totally) disagree	23	18.5	46.6	91.0	22	6.1	22.7	41.8	27	9.8	36.4	75.2
Neutral	29	25.4	47.5	56.8	25	11.2	18.6	44.2	22	8.5	28.0	47.5
I agree	91	22.5	41.5	67.7	53	10.3	20.4	34.6	66	20.0	30.0	50.0
I totally agree	96	22.0	44.6	78.6	71	9.4	22.6	53.8	121	14.4	32.3	56.5
*P*-value				0.6896				0.7581				0.8067
**“It affects me to see a cow in pain.”**
I disagree/neutral	17	45,5	82,7	100	5	10.1	28.6	87.7	7	10.4	36.4	75.2
I agree	90	25,5	43,1	74.4	58	12.6	20.9	34.6	60	15.2	28.3	48.4
I agree totally	132	19,7	41,1	70.1	108	9.8	20.7	48.0	169	14.4	30.2	56.5
*P*-value				0.0073				0.8418				0.6331
**“My job as a farmer puts weight on me.”**
I totally disagree	34	17.6	37.2	77.9	24	7.6	14.1	23.6	67	10.4	24.0	50.0
I disagree	98	26.6	49.6	77.9	52	11.4	21.6	46.8	59	16.7	34.1	60.0
Neutral	52	16.7	32.7	62.1	34	12.6	22.7	49.7	53	14.6	29.9	50.0
I agree	42	20.0	43.9	70.5	47	10.1	21.5	43.4	50	19.4	33.3	64.9
I totally agree	11	36.2	45.5	67.2	15	7.1	22.4	51.8	8	14.3	19.2	57.1
*P*-value				0.0741				0.5854				0.3681
**Claw health as a breeding goal**
No	70	18.3	44.5	86.7	58	6.9	15.6	37.9	120	15.0	32.5	66.3
Yes	171	24.5	42.9	70.8	183	13.2	28.3	53.0	116	14.7	28.7	46.9
*P*-value				0.8015				0.0028				0.1633

a *Two year education at an agricultural college or “Meister”-degree*.

Farmers who stated that they were totally satisfied with the health of their herds had lower FDI than the other farmers (Table 6). This finding was consistent across all the three regions. In the north, farmers who stated that they were (totally) unsatisfied with the health of their herds had the highest FDI. However, in the east and south, farmers who were neutral or agreed with the statement, “I am satisfied with the health of my herd,” had the highest FDI (Table 6). Farmers who stated claw health to be a major breeding goal had higher FDI (east and south; Table 6).

No statistically significant associations were consistently apparent between emotionality, agreeableness, conscientiousness, and openness. However, in the south, more extraverted farmers had lower FDI (Table 7; *p* < 0.0001).

**Table 7:** Spearman's correlation analyses: Farmers' Detection Index and farmers' personality traits (HEXACO).

	**North**			**East**			**South**		
	***N***	**SCC**	***P*-value**	**N**	**SCC**	***P*-value**	***N***	**SCC**	***P*-value**
Emotionality	191	0.03	0.6672	175	0.02	0.7931	231	0.04	0.5088
eXtraversion	191	−0.03	0.6995	175	−0.13	0.0752	231	−0.26	<0.0001
Agreeableness	191	−0.13	0.0813	175	0.04	0.5627	231	0.05	0.4865
Conscientiousness	191	0.02	0.7713	175	0.02	0.7885	231	0.04	0.5869
Openness	191	0.03	0.6707	175	0.12	0.1090	231	−0.11	0.0877

*SCC, Spearman's Correlation Coefficient*.

## Discussion

This study shows that the majority of farmers underestimated the proportion of lame cows within their herds. Factors related to farmers' mindset tended to have a stronger association with FDI than factors from field claw health management or farm structure.

### Study Design

The study was conducted in three regions differing in herd size, performance, and management. Because of the sufficient sample size, it was possible to analyze these regions separately. In our view, it was mandatory to analyze the regions separately due to the aforementioned differences. The separate analyses increased the validity of the results and showed similarities and differences concerning the prevalence, estimation of the farmers, and associations between FDI and different factors.

Participation in the study was voluntary. Therefore, the prevalence of lame cows might be biased compared to that of the underlying target population. Although selection was at random from a preformed list of farmers, farmers had to answer to the invitation by contacting the study team. Therefore, potentially more proactive and open farmers or those with better-managed dairy farms may have been enrolled and visited, which could have resulted in an underestimation of the true lameness prevalence. On the contrary, voluntary participation may have motivated farmers with problems concerning their herds' health to participate (38).

Historically, locomotion scoring approaches have been implemented to record the characteristics of dairy cow gait and to subsequently classify cows as lame and not lame. Among these approaches, Sprecher et al. (24) presented the most widely used locomotion scoring systems that are based on posture and gait (38, 39). Evidence has suggested that slight locomotive aberrations, such as an arched back (score 2; “mildly lame”), may lead to marked consequences for production level and animal welfare (24, 40). However, most studies relying on the Sprecher score classify cows as lame when they receive a score ≥ 3. Therefore, we followed this categorization.

For tie stall systems, the SLS (25) was implemented and, in alignment with previous work (41, 42), a cow was classified as lame if two out of the four indices of the SLS were recorded. Lameness detection in tie-stall facilities is more challenging than in loose housing systems, as cows cannot be observed during locomotion. Furthermore, Leach et al. (25) observed a moderate sensitivity of the SLS (0.54–0.77) compared with the Sprecher score; on average, the prevalence of lameness was underestimated by 27% (11–37%) in their study when assessing cows in tie stalls. This needs to be taken into consideration when interpreting our results as well, and as a consequence, the prevalence of lame cows in tie stall operations might have been underestimated. In the regions north and east only a minor proportion of cows was scored using the SLS (north: 1.4%; east: 0.3%), but in the region south 16.6% of the cows were scored using the SLS.

Three observer training sessions were carried out during the study to reduce potential observer bias. Only moderate agreement was achieved when comparing the ordinal ratings of the observers. Aberrance of single observers, but no aberrance of the team of a region was observed. Therefore, a systematic bias leading to a higher or lower prevalence in one region compared to the other was unlikely. A comparison of the classification (severe) lame–not lame was not conducted. This would probably have resulted in a better agreement. However, the moderate agreement despite the use of SOPs and training shows that the classification of lameness is challenging. This might be one reason for the underestimation by the farmers and points additionally toward the need of widely applying more objective lameness detection techniques (e.g., automatic lameness detection systems).

Farmers might have stated a lower number of lame cows due to social desirability. Social desirability bias describes the tendency to state things that place the interviewee in a favorable light (43). The social desirability bias consists of two separate factors: “Other-deception” describes that a person purposely misrepresents the truth as a form of impression management motivated by a desire to avoid evaluation. “Self-deception” occurs when a respondent actually believes a statement to be true of himself or herself, even though it is inaccurate (43). As the FDI describes the difference between the situation systematically assessed by trained researchers and the perception of the farmers, it describes nothing other than the bias of social desirability; the question is how strong the influence of self-deception and other-deception was. Concerning the influence of other-deception, on one hand, farmers might have consciously stated a lower percentage of lame cows as they were aware that they were talking to a veterinarian. Veterinarians are experts on the health of animals. Therefore, farmers might have felt controlled or might have been under pressure to state a lower number than they might have stated in another context. On the other hand, it can be hypothesized that farmers might also have stated a higher number of lame cows because of the professional background of the interviewers. Farmers may trust veterinarians more than the general population, as they expected them to know about the problems in dairy herds. Moreover, farmers were aware that locomotion scoring was performed. Therefore, they might have been motivated to provide a preferably correct estimation.

To speak from our experience within this study, most farmers reacted honestly concerned or shocked when they were handed a list of lame cows at the end of the farm visit. Therefore, we expect that the FDI was less influenced by other-deception than by the fact that farmers were actually not aware of the lameness situation (self-deception).

### Lameness Prevalence and Farmers' Awareness

The median within-farm prevalence of lame (≥3) and severely lame (≥4) cows, as determined by study veterinarians, was similar or even higher compared with most studies from different areas of the world addressing this issue in the past 5 years (Table 8). Farmers' estimations were high compared to those in recent studies (Table 8). In previous studies, farmers were aware of an even lower proportion of lame cows [Table 8; (28, 37, 44)]. This finding may be related to the fact that farmers in the present study were explicitly asked to include those cows that were mildly lame in their estimations. This might also be the reason, that in total 62 farmers overestimated the proportion of lame cows. Half of these farmers had a good estimation of the lameness situation but nine farmers overestimated the prevalence by factor two or more. Most of these farmers had <40 cows, so that one or two had a great impact on the prevalence.

**Table 8:** Studies published during the last 5 years reporting the prevalence of lame cows.

**Authors**	**Average within herd prevalence of…**						
	**…lame cows assessed by researchers**	**…severely lame cows assessed by researchers**	**...lame cows estimated by farmers**	**Number of farms**	**Number of cows**	**Country**	**Main housing system**
Jensen et al. current data set	29.7%	11.4%	11.6%	751	84,998	Germany	misc.
Denis-Robichaud et al. (33)	36.9%	n/a	25.3%	93	n/a	Canada	tie stalls
Beggs et al. (34)	3.2%	n/a	0.8%	50	19,154	Australia	pasture
Jewell et al. (35)	20.7%	n/a	n/a	40	2,719	Canada	freestalls
Jewell et al. (35)	15.3%	n/a	n/a	33	1,346	Canada	tie stalls
Bran et al. (17)	n/a	14.4%	6.5%	44	1,633	Brazil	pasture
Griffiths et al. (9)	31.6%	3.7%	n/a	61	14,700	UK	freestall
Cutler et al. (11)	22.2%	n/a	9.0%	237	n/a	Canada	misc.
Adams et al. (36)	9.8%	2.6%	n/a	184	22,042	USA	misc.
Ranjbar et al. (37)	19.1%	n/a	5%	63	18,960	Australia	pasture

*n/a, not available (not assessed or not reported); misc. , miscellaneous*.

Some authors assume that farmers experience only severely lame cows as “lame” and overlook mildly lame cows (13, 18). In this study, no substantial or good agreement was detected between FP and VP_severe. The agreement between FP and VP_severe was only slightly higher than that between FP and VP in the east and south. Therefore, the hypothesis that farmers rate severely lame cows as “lame” and overlook moderately lame cows was not confirmed. As the consequences of lameness for animal well-being are a subject of discussion these days, improvement of claw health is of priority for the farmers. Nevertheless, a mean FDI between 36 and 56% cannot be regarded as satisfying. Keeping in mind the high prevalence of lameness in cows, the question is what has to be done to raise awareness.

Recently, research in veterinary consultation and communication has revealed that motivational interviewing is a promising approach to help farmers change their behavior and improve their herd health status (45, 46). Motivational interviewing is linked to the transtheoretical model of behavior change (47). The transtheoretical model describes the stages and processes of change to explain health behavior changes (48). In the first stage, precontemplation, people are not likely to change their behavior in the near future. They are often not well-informed about the problem or tried to improve the situation unsuccessfully and became demoralized. Those persons tend to avoid engaging with this problem and may be perceived as “unmotivated” or “resistant.” One of the factors motivating a person to change is the perceived severity of the disease (49). As lameness was strongly underestimated, it can be assumed that most farmers were not likely to take any measures in the near future and were in the stage of precontemplation. In the transtheoretical model, three cognitive processes of change are described that can initiate the stage of contemplation and the intention to take measures. These processes of change are consciousness raising (for example, feedback, new information, confrontation, or media campaigns), dramatic relief (for example, traumatic experiences such as the loss of a valued cow), and self-re-evaluation (by becoming aware that a certain problem may be caused by oneself). Santman-Berends et al. (50) showed that the phase of awareness plays a crucial role in herd health problems. Keeping in mind that most farmers underestimate the prevalence of lameness in dairy cows, client-centered interventions, including the transtheoretical model and motivational interviewing, might be helpful in controlling lameness in dairy cows. Therefore, further research exploring the effects of the transtheoretical model and motivational interviewing on lameness awareness and the implementation of on-farm control is required.

### FDI and Farm Characteristics

Concerning the housing system, we expected farmers housing their cows in tie stalls to have a lower FDI, as observed by Cutler et al. (11). In tie stalls, lameness detection might be impaired, as cows can only be seen in movement when they have access to pastures. Only in the south did an adequate number of farmers kept their cows tied most of the time. The FDI was similar between farmers keeping the cows in barns with cubicles and those keeping their cows tied. This can be due to the fact that in tie stalls, the method of lameness detection is not as sensitive as in free stalls. Another reason could be that lameness is usually a longlasting condition and most cows kept in tie stalls in Germany also have grazing periods. During these, farmers can observe the cows' locomotion daily when collecting them for milking.

In the north, farmers who kept their cows in alternative housing systems, such as straw pens, full-time pasture, or mixed systems, had higher FDI than those who kept their cows in free stall barns. However, this finding was not statistically significant. Farmers who operated according to organic principles had a higher FDI than farmers who operated their farms conventionally in the regions north and south. In organically managed farms, lower lameness prevalence was observed (8, 51). Farmers who decide to manage their farms according to organic principles might develop more activities to meet animal welfare needs (52). Our findings suggest that as the awareness of animal health and welfare may be generally higher in organic farming, this might also include higher awareness of claw health.

Concerning herd size, one may expect a lower FDI in larger herds, as the single lame cow may be overlooked (13). Only in south, a weak association between FDI and herd size was observed. Comparing the three regions, FDI was lower in the region east, where herds were larger. However, the highest FDI was observed in the north and not in the south. Fabian et al. (16) observed no association between the FDI and herd size.

Fabian et al. (16) observed higher estimated lameness incidences in dairy herds with a higher average milk yield per cow. In our study, no association was detected between the average milk yield and FDI. On the one hand, the risk for lameness is higher in high producing herds. On the other hand, farmers of high producing herds may have a higher awareness toward herd health problems.

In the east, farmers who admitted to culling a higher percentage of cows due to lameness had a higher FDI. This finding implies a better awareness of the lameness status when the farmer is aware of the reasons for culling or, rather, a higher overall awareness of lameness and its consequences. However, this finding was not significant in the other regions. In the east, the documentation of culling reasons might be better than in the other regions due to a more professional organization and the use of software for surveillance of herd health.

### FDI and Claw Health Management

There were no associations between claw health management and farmers' awareness of lameness (Table 5), even though one might expect that farmers who experience lameness as a problem might take measures to control it.

Only 17% (north), 34% (east), and 1% (south) of the farms participated in a VHHMP that included claw health. In the region with the highest lameness prevalence (east), more farmers participated in the VHHMP than in the other two regions. Moreover, FDI was not higher on farms participating in these programs or in any other VHHMP (Table 5). We expected that awareness might be higher when a consultant provided regular feedback. Leach et al. (28) observed an increasing awareness of claw health in farmers who took part in a project with expert feedback on claw health issues on a regular basis. As a consequence, lameness prevalence decreased in farms with an initial lameness prevalence of up to 35%. These contrasting results show that further research is needed on the effect of regular feedback on the development of awareness and lameness prevalence as well as on the type of feedback that could initiate change.

The majority of farms performed claw trimming, on average, twice a year per cow (Table 5). There was no association between the frequency of claw trimming and FDI. We expected to have a higher FDI in farms with a higher frequency of claw trimming for two reasons: first, lame cows (and their claw lesions) might be detected during claw trimming, and second, farmers with a higher awareness might perform claw trimming more often to fight this problem. Even if farmers accurately estimate lameness prevalence, that alone does not equate to action being taken (53). Moreover, no association was found between the person trimming the claws and FDI. On the one hand, many professional claw trimers give feedback to the farmers concerning the prevalence of certain diagnoses and might be more aware of lameness due to their professional training. On the other hand, one might expect a higher FDI if the farmers performed the claw trimming themselves, as they might be paying more attention to the claws and the lame cows.

Most farmers stated that they control the presence of lame cows when fulfilling their routine tasks in the stable. Only a few farmers performed lameness detection as a separate task (Table 5). This finding is in accordance with a study by Cutler et al. (11). Horseman et al. (18) showed that farmers feel that existing methods are adequate to detect lameness and that locomotion scoring would add nothing more to their detection process. Moreover, visual locomotion scoring systems are time-consuming and require knowledge and experience (54). The data need to be processed to provide maximal benefit. None of the farmers made use of an automated lameness detection system to support the early detection of lameness (3, 55). Farmers who do not experience lameness as a major problem in their herds may not be motivated to invest money in an automated lameness detection system. This might be one reason why these systems are not widely used in Germany.

### FDI and the Attitude and Education of the Farmers

The only significant association in all three regions was the association between herd health satisfaction and FDI. Farmers who stated that they were totally satisfied with the health of their herds had a lower FDI than the other farmers (Table 6). These farmers seemed to be unaware of problems or might not be willing to admit that they had problems with their herd's health. In the north, farmers who stated that they were (totally) unsatisfied with the health of their herds had the highest FDI. This finding appears logical, as these farmers seemed to be aware of and able to admit the deficiencies concerning their herd's health. Surprisingly, in the east and south, farmers who were neutral or agreed with the statement, “I am satisfied with the health of my herd” had the highest FDI (Table 6). Despite better awareness of the lameness situation, these farmers were generally okay or satisfied with their herd's health. These farmers might be focusing on acute diseases such as severe cases of mastitis or hypocalcemia and not on chronic or progressive diseases such as lameness when thinking of herd health. Moreover, a high FDI does not imply a high prevalence of lameness.

Education and attendance at technical seminars were not as strongly associated with FDI, as one might expect. Farmers with a university degree in agriculture had higher FDI in the north and south. In the east, farmers with no agricultural education had lower FDI than other farmers. However, these findings were not statistically significant (Table 6). Further research is needed concerning the association of education and lameness detection.

To assess empathy towards cows, the farmers had to state their agreement with two sentences (“I have an emotional relationship with my cows.” and “It affects me to see a cow in pain.”). Surprisingly, those farmers who stated not to agree or those who felt neutral toward the second statement had the highest median FDI in all three regions compared with those who agreed or those who agreed completely (Table 6). We can only speculate that these farmers might have been the most honest ones and were less biased by social desirability, or they had a more rational and analytic mindset toward their cows. Also, some farmers who know that lameness is an animal welfare issue might not be able to admit that their herd also has many lame cows because it poses a significant burden for them for that no quick and easy solution exists. In contrast, the study of Bruijnis et al. (56), farmers who rated foot disorders as important for animal welfare were more motivated to take actions for foot health improvement.

### FDI and Farmers' Personality

To our knowledge, this is the first study examining awareness of lameness in dairy cows and traits of the farmer's personality, even though farmer's personality is known to influence dairy cattle health and welfare (21). More extraverted farmers tended to have lower FDI. The reason for this finding can only be assumed. Extraverted persons have a higher self-acceptance and are happier in life (57). Therefore, they may be carefree and tend to experience lameness not as much of a problem. Also, extraverted farmers are more likely to have positive relationships with others (57). Thus, the correlation between extraversion and the FDI may be due to other-deception.

Regrettably, Honesty–Humility was not assessed in the shortened version of the HEXACO-questionnaire due to reasons of acceptance. The study was designed to assess cattle health, housing, and management. Indeed, some farmers reacted perplexed to the questionnaire handed out at the end of the interview containing statements such as “I enjoy contemplating paintings.” However, the assessment of Honesty–Humility would possibly have provided interesting insights.

Conscientiousness is a strong predictor concerning desirable health behavior (58). O'Kane et al. (59) reported that sheep farmers with a high score in conscientiousness reported to have fewer lame sheep. In this study, no associations between conscientiousness and the awareness of lameness were detectable (Table 7).

### Conclusions

Our study revealed that lameness remains a major problem in many dairy herds in Germany. However, the large variance showed that some farmers succeeded in lameness control and prevention. The prevalence estimated by farmers was substantially lower than that assessed by veterinarians. The poor agreement between farmers' estimated lameness prevalence and observers' results clearly demonstrate the need to support farmers in raising awareness of lameness.

The analyses concerning the associations with the FDI gave new insights into the mindset of farmers concerning the awareness of lameness; farmers managing their farm according to organic principles or using housing systems with improved cow comfort had a better awareness of lameness. Contrary to other studies, the hypothesis that in larger herds, individual cows receive less attention could not be confirmed. Moreover, there were no statistically significant associations between claw health management and FDI, leaving the question of how the awareness and implementation of actions are linked. A weak association between education and FDI could be detected, indicating that information is helpful in raising awareness of lameness. The results indicate that poor awareness of lameness was linked to the farmers' attitude and personality. Therefore, new approaches concerning the consultation regarding lameness control, such as the use of Motivational Interviewing, may well be useful in the future.

## Data Availability Statement

The raw data supporting the conclusions of this article will be made available by the authors, without undue reservation.

## Ethics Statement

Ethical review and approval was not required for the study on human participants in accordance with the local legislation and institutional requirements. The patients/participants provided their written informed consent to participate in this study. Ethical review and approval was not required for the animal study because no painful interventions have been made. This was in accordance with the local legislation and institutional requirements. Written informed consent was obtained from the owners for the participation of their animals in this study.

## Author Contributions

KJ, AS, SW, and AO visited the farms and collected the data. KJ and RM analyzed the data and discussed it with AO, MF, KM, and AS. AC developed the assessment of the farmers' personality and attitudes. KJ wrote the first draft of this work. All authors were involved in the planning of the study, revised the article critically, and contributed substantial ideas. All authors contributed to the article and approved the submitted version.

## Funding

Funding of this project was provided by the Federal Ministry of Food and Agriculture and Federal Office for Agriculture and Food, grant numbers 2814HS006 (University of Veterinary Medicine Hannover), 2814HS007 (Freie Universität Berlin), and 2814HS008 (Ludwig-Maximilians-Universität Munich).

## Conflict of Interest

The authors declare that the research was conducted in the absence of any commercial or financial relationships that could be construed as a potential conflict of interest.

## Publisher's Note

All claims expressed in this article are solely those of the authors and do not necessarily represent those of their affiliated organizations, or those of the publisher, the editors and the reviewers. Any product that may be evaluated in this article, or claim that may be made by its manufacturer, is not guaranteed or endorsed by the publisher.
